# Fish and Fish-Based Products for Nutrition and Health in the First 1000 Days: A Systematic Review of the Evidence from Low and Middle-Income Countries

**DOI:** 10.1093/advances/nmac102

**Published:** 2022-09-27

**Authors:** Kendra A Byrd, Jacqueline Shieh, Stephanie Mork, Lauren Pincus, Lydia O'Meara, Molly Atkins, Shakuntala H Thilsted

**Affiliations:** WorldFish, Bayan Lepas, Penang, Malaysia; WorldFish, Bayan Lepas, Penang, Malaysia; St Catherine University, St Paul, Minnesota, USA; WorldFish, Bayan Lepas, Penang, Malaysia; WorldFish, Bayan Lepas, Penang, Malaysia; Natural Resources Institute, University of Greenwich, Chatham Maritime, Kent, UK; WorldFish, Bayan Lepas, Penang, Malaysia; International Development Department, University of Birmingham, Birmingham, UK; WorldFish, Bayan Lepas, Penang, Malaysia

**Keywords:** fish, aquatic foods, pregnancy, breastfeeding, complementary feeding, maternal diets, IYCF practices

## Abstract

Fish provide essential nutrients for the critical window of growth and development in the first 1000 d of life and are thus an attractive option for inclusion in nutrition-sensitive and nutrition-specific programming. We conducted a systematic review of the evidence for fish and fish-based products for nutrition and health outcomes during the first 1000 d of life in low- and middle-income countries, searching the peer-reviewed and gray literature from 1999 to 2020. Databases included PubMed, Web of Science, Embase, ProQuest, and the Clinical Trials repository. Our search returned 1135 articles, 39 of which met the inclusion criteria. All studies were dual evaluated for risk of bias. Of the included studies, 18 measured maternal health and nutrition outcomes and 24 measured infant/child outcomes (3 measured both). Our search uncovered 10 impact evaluations, all of which measured consumption of fish or fish-based complementary food products in children aged 6–24 mo. We did not find strong evidence for fish consumption in children improving child growth from the impact evaluations; however, the studies were highly heterogeneous in their design and likely underpowered to detect an effect. Results from observational studies were mixed but provided evidence that adding fish to maternal and child diets is associated with improved nutrition outcomes, such as reducing the risk of anemia and improving vitamin D status. Given the nutrient richness of fish and the fact that production is often more environmentally friendly as compared with other animal source foods, more robust evidence is needed on the role of fish consumption in nutrition interventions to inform policy and programming recommendations in low- and middle-income countries.

## Teaser

**Statement of Significance**: This is the first systematic review providing an overview of the current evidence to date on the role that fish and fish-based products play in addressing the burdens of malnutrition in women and children. Whereas evidence is emerging that fish and fish-based products improve health and nutrition outcomes, more trials with robust study designs are needed to clearly elucidate the benefits.

## Introduction

The first 1000 d of life (in utero and age ≤2 y) is the most critical period for cognition, growth, and development (1). Nutrient deficiencies in the first 1000 d can lead to serious adverse health and economic consequences later in life, especially for children in low- or middle-income countries (2).

Evidence suggests that fish consumed in the first 1000 d—first by pregnant and lactating women and then by infants and children during the complementary feeding period (when the child is 6–23 mo old and foods complement breastmilk)—has the potential to improve nutrition and health outcomes for these vulnerable populations. During the first 1000 d, nutrients such as iron, zinc, calcium, iodine, vitamin B-12, vitamin A, essential fatty acids, and protein, which are often found in fish, are critical for healthy pregnancies and for the optimal growth and development of young children (3, 4). Fish of all sizes are higher in essential ω-3 fatty acids when compared with other animal source foods (ASFs), and small fish have a higher micronutrient concentration than large fish (4), primarily because they are consumed whole. Furthermore, when small fish-based products are dried and powdered, the iron, zinc, calcium, and fatty acid concentrations are comparable to commercially produced complementary food supplements, such as small-quantity lipid-based supplements (5). Small fish also have a high reproductive rate and, in some places in sub-Saharan Africa, are sometimes not fished to their maximum sustainable yield (6). These characteristics make fish, especially small fish, an appropriate component of programs targeting malnutrition. However, a review of the evidence is needed to inform policy makers and other stakeholders regarding the importance of fish and fish-based products for addressing malnutrition.

Prior systematic reviews on fish consumption have largely focused on high-income countries (7, 8) or ASFs in general (9–11). To our knowledge, no studies have systematically reviewed the evidence on how whole fish or fish-based product consumption during the first 1000 d of life affects the nutrition and health of pregnant and lactating women and infants from low- and middle-income countries. In this systematic review, we collate findings on fish and fish-based products and the role that they play in nutrition and health outcomes of pregnant and lactating women and children <2 y of age to better inform future nutrition interventions for women of reproductive age and children in low- and middle-income countries.

## Methods

The PRISMA guidelines informed this systematic review (12). To assess the available literature investigating fish and fish-based products and their relationship to nutrition and health outcomes in the first 1000 d, a protocol was developed outlining the systematic review aims, search strategy, and eligibility criteria (Table 1). We brainstormed specific search terms that encompassed the diverse array of fish and other aquatic foods and terms that cover the first 1000 d (from pregnancy to ≤2 y of life). This led to the following search terms:

(fish OR seafood OR prawns OR shrimps OR aquatic animals OR fish powder OR fish-based products) AND (complementary feeding OR complementary food OR mothers OR young children OR infants OR pregnan* OR lactat*) AND (low-income countr* OR middle-income countr* OR developing countr* OR Africa OR South East Asia OR South America OR Pacific Islands).

**TABLE 1:** Inclusion and exclusion criteria to select studies for the systematic review exploring the relationship between fish and fish-based products and nutrition and health outcomes in low- and middle-income countries^1^

Criterion	Include	Exclude
Publication type	Peer-reviewed articles, dissertations	Qualitative studies, ethnographic studies, case studies, unpublished abstracts and reports
Publication years	1999–2020 (dates originally selected to capture previous 20 y but 2020 was added post hoc)	
Population	Pregnant and lactating women, children aged 0–24 mo, and any population that included people in this life stage	Studies that had no participants in this life stage
	Low- and middle-income countries as classified by the World Bank for the fiscal year 2020	High-income countries as classified by the World Bank for the fiscal year 2020
Language	English	Papers not written in English
Study type	Quantitative studies, including randomized controlled trials, longitudinal studies, and cross-sectional studies	Descriptive trials of diets (no nutrition or health outcomes measured)
Intervention/exposure	The exposure of interest for this study was the consumption of fish or fish-based products (in frequency or amounts)	Nutraceuticals (i.e., fish oil)
	Studies that included fish consumption alongside other foods must have included data on fish consumption as a subgroup	Fish intakes at the household level
Comparator	When a comparator was used, the comparator could include complementary foods that did not contain fish	
Outcome measures	Maternal and infant anthropometrics (e.g., LAZ/HAZ, WAZ, WHZ, MUAC)	Food safety articles investigating pathogens in fish
	Nutritional status determined by biochemical means (e.g., serum tests), such as macronutrient (e.g., fatty acid) or micronutrient concentrations, or outcomes related to micronutrients (e.g., hemoglobin concentrations)	Articles examining fish intake on the incidence of allergies
	Other health and morbidity outcomes, such as mental health, respiratory infections, or diarrhea	Articles focused on diets and not nutritional status or health outcomes
		Articles examining fish intake on breastmilk concentrations

1 HAZ, height-for-age *z* score; LAZ, length-for-age *z* score; WAZ, weight-for-age *z* score; WHZ, weight-for-height *z* score.

Given that the term “aquatic foods” is a relatively new term used by researchers and policy makers, we did not include it in our search. However, if found, studies were included on other aquatic foods (e.g., snails).

In July 2019, we reviewed the literature published in the previous 20 y (1999–2019) and updated the search to an additional year in July 2020 (July 2019–July 2020). We included the following databases in our search: PubMed, Web of Science, Embase, and ProQuest, as well as a hand search of the Clinical Trials repository (clinicaltrials.gov). We also hand searched gray literature sources such as the International Food Policy Research Institute's institutional repository. We reviewed the reference lists of systematic or nonsystematic reviews for studies that met our criteria. The titles and abstracts of all search results were independently screened by one author (KAB) and then by another (JS). Studies that met the inclusion and exclusion criteria were retained, and duplicates were removed. Any discrepancies that involved retaining full-text articles were discussed and resolved between the screeners and, when necessary, a third author. Details of the full-text articles regarding study design, population, setting, interventions/exposures, sample size, analysis methods, outcomes measured, and results were extracted in duplicate into a predefined piloted table (Tables 2a–c). Data extraction was conducted by SM and reviewed by at least 1 other author for accuracy.

Critical appraisals were conducted independently by 2 authors (JS and SM). Disagreements in ratings were discussed and revised between the appraisers and a third author when necessary. Studies were rated as having low, medium, or high risk of bias based on the aspects on their study design and how well the methods and findings were reported. Several critical appraisal tools were used due to the range of study designs in this review. The revised Cochrane risk-of-bias tool for randomized trials (13) was used for randomized controlled trials to assess the effect of assignment to interventions. As all interventions were food based, questions on concealment of participants and people delivering interventions were not considered (Nos. 2.1 and 2.2). Yet, questions were assessed as related to researcher and/or outcome assessor concealment prior to analysis (Nos. 1.2 and 4.3). The ROBINS-I (Risk of Bias in Non-Randomized Studies of Interventions; 14) was used for nonrandomized controlled trials. Questions from the ROBINS-I tool pertained to confounding, participant selection, intervention classification, deviations from intended interventions, missing data, outcome measurement, and reporting bias (14). For cohort (15) and case–control (16) studies, the respective Critical Appraisal Skills Program (CASP) checklists were used. The CASP checklists assessed participant recruitment and selection, exposure measurement, confounding, and internal consistency of results. The CASP Cohort Checklist also included questions on subject follow-up (15), whereas the CASP Case–Control Checklist included questions regarding treatment of cases and controls as well as treatment effect size and measurement (16). The Appraisal Tool for Cross-Sectional Studies (17) was used for cross-sectional studies. Questions assessed sample size justification, selection bias, exposure and outcome measurement, confounders, nonresponse bias, and internal consistency of results (17). Given the large breadth and heterogeneity in interventions, exposures, comparators, and outcomes, all included studies were narratively synthesized.

## Results

### Overview of included studies

**FIGURE 1:**
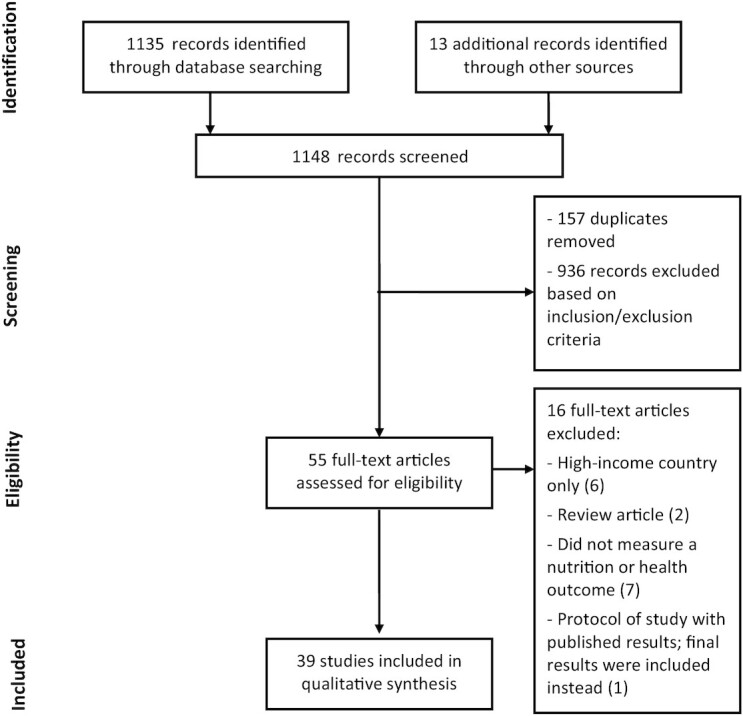
PRISMA flow diagram for the screening and inclusion of publications in this systematic review that investigated the relationship between fish and fish-based products and nutrition and health outcomes in low- and middle-income countries.

The total number of titles and abstracts screened per the inclusion/exclusion criteria was 1148 (Figure 1). Fifty-five full texts were ultimately assessed for eligibility, of which 16 were excluded, resulting in the inclusion of 39 studies. The main reasons for exclusion were that the participants were from a high-income country or the study did not measure a nutrition or health outcome. For example, several studies examined the nutrient content of a fish-based product, rather than how the fish-based product affected the nutrient status of a person. Most studies were cross-sectional (*n* = 21), followed by randomized controlled trials (*n* = 10), cohort studies (*n* = 6), case–control studies (*n* = 1), and nonrandomized controlled trials (*n* = 1).

### Study details and demographics

The 39 studies were conducted across 16 low- and middle-income countries. The studies can be divided into observational studies (*n* = 29) (Table 2) and impact evaluations (*n* = 10) (Table 3). Of the 39 studies, 14 were in Eastern and Southern Africa (18–31), 5 in West and Central Africa (32–36), 10 in Southeast Asia (37–46), 4 in South Asia (47–50), and 5 in Latin America and the Caribbean (all of which were in Brazil) (51–55). One study included data from 46 low- and middle-income countries (56). The study populations in most studies were infants (18, 20, 22–25, 27, 29, 30, 33–42, 44, 47, 51–53, 56–58), followed by pregnant and/or lactating women (19, 32, 46, 48, 49, 53, 55) and mothers of young children (19, 27–30, 33, 37, 50, 51, 53). For the 10 impact evaluations, study durations ranged from 2 to 59 mo. Among the 8 longitudinal studies, Pinto et al. (54) had the smallest sample size (*n* = 146) and shortest study duration (9 mo). The largest sample size was >1 million (49) and the longest study duration was 5 y (51, 53). The sample sizes for the 21 cross-sectional studies ranged from 60 participants (41) to 130,432 (56). Tables 2 and 3 give a breakdown of study designs, exposures, and measured outcomes.

**TABLE 2:** Observational trials included in a systematic review exploring the relationship between fish and fish-based products and nutrition and health outcomes in low- and middle-income countries^1^

Author, year, study location	Study objective	Study characteristics	Data analysis methods	Outcomes measured	Key findings	Conclusions	Risk of bias
Aiga 2009 (18)Malawi	To investigate the associations between fish farming and nutritional status	Cross-sectional study:66 children aged 6–59 mo in each type of household (fish farming and non-fish farming)	Bivariate and multivariate analyses	• Stunting• Underweight• Wasting	A significantly lower prevalence of severe and moderate underweight was found among the children in fish-farming households vs. non-fish-farming households, likely through the pathway of increased income from fish farming	Household fish farming may have indirectly contributed to lower prevalence of underweight by increasing household purchasing power to obtain a wider variety of foods, such as oil and fats	Low
Angkasa 2017 (37)Indonesia	To test the associations between maternal fatty acid intake (total fat, total ω-3, EPA, DHA, and ALA) and birth weight	Cross-sectional study:282 pregnant women aged 19–40 y with gestational age >32 wk	Multiple linear regression	• Birth weight• Head circumference• Birth length	• Newborns of mothers with lower ALA intakes had a lower birthweight than those with higher ALA intakes.• Total fat, total ω-3, DHA, and EPA intakes were not associated with birth weight.• Total fat and ω-3, EPA, DHA, and ALA were not associated with birth length or head circumference	Promoting ω-3, in particular ALA, may enhance optimal infant weight in certain contexts, such as urban areas in Indonesia	Medium
Anlaakuu 2017 (32)Ghana	To determine the factors associated with anemia in pregnancy, such as previous pregnancies, malaria infection, and the frequency of consumption of fish/snails among pregnant women who sought prenatal care	Cross-sectional study:316 pregnant women aged 15–45 y at first and most recent antenatal care visits	Bivariate and multivariate statistical analysis	Anemia	• Most women consumed fish or snails ≥2/wk, and this was associated with a reduced prevalence of anemia.• However, there was no association between snail/fish consumption and anemia in the adjusted model	Improving dietary intakes and regular monitoring of hemoglobin concentrations during pregnancy could help reduce anemia in this vulnerable population	Medium
Bosha 2019 (19)Ethiopia	To investigate the association between dietary diversity and anthropometric status of mother–child pairs during varying harvest seasons	Cross-sectional study:578 mother–child pairs: mothers of reproductive age and children aged 24–59 moData on dietary diversity and anthropometric status were collected during postharvest dry season and lean wet season	Multivariate logistic regression	• BMI of nonpregnant women, MUAC of pregnant women• WAZ and WHZ of children	• Maternal and child anthropometric measures decreased during the lean wet season.• ASF consumption was not significantly associated with anthropometric status in either the mothers or children	ASF intake for mothers and children in Ethiopia is low in wet and dry seasons, and there is a decline in anthropometric status for mother–child pairs, specifically in the lean wet season	Medium
Cunha 2018 (51)Brazil	To test association between the frequency of maternal fish intake on child anthropometry	Secondary analysis from longitudinal study:1433 women and children followed from birth to 5 y	Linear mixed effects model	At birth, 6, 24, and 59 mo:• LAZ/HAZ• WAZ• WHZ	Maternal fish intake was not significantly associated with LAZ, HAZ, WAZ, or WHZ after model adjustments	High consumption of freshwater fish during pregnancy was not associated with anthropometric measurements of children followed from birth until age 5 y	Medium
Dei-Tutu 2020 (33)Ghana	To investigate the hypothesis that maternal iodine deficiency affects infant thyroid function	Cross-sectional study:250 mother–infant pairs (newborn)	Chi-square test	Infant iodine status	• Intake of iodine from iodized salt, bouillon cube, and seafood was generally high, though 30% of mothers in this study were iodine deficient.• Maternal seafood intake was not associated with infant TSH	More research is needed to establish norms for TSH concentrations in infants and describe the relationship between maternal iodine status and infant thyroid function	Medium
Hawlader 2019 (47)Bangladesh	To identify the lifestyle and nutrition factors associated with vitamin D deficiency among Bangladeshi	Case–control study:• 198 healthy children• 198 children with vitamin D deficiency	Univariate and multivariate logistic regression	Serum vitamin D concentrations	Absence of marine fish consumption was associated with a vitamin D deficiency in Bangladeshi children	The key determinants of vitamin D deficiency among Bangladeshi children include dietary habits and lifestyle factors.	Medium
	children aged 1–13 y					Vitamin D supplementation and education on diet (including marine fish) and lifestyle should be considered to decrease vitamin D deficiency	
Headey 2017 (56)Global	To test association between ASF (including fish consumption separately) and stunting prevalence	Secondary data analysis from cross-sectional surveys:117,887 children aged 6–23 mo in 46 low- and middle-income countries	Probit model	Stunting prevalence	• All ASF intakes (milk, eggs, meat, fish) were associated with a reduced stunting prevalence.• Consuming multiple ASFs had a stronger association with reduced stunting than any single ASF	ASF, including fish, is beneficial to reduce stunting, but many developing countries have limited access to ASF because of cost and supply chain issues. Increasing access to ASF would be beneficial for child growth	Low
Kaimila 2019 (23)Malawi	To test associations of the type of protein and quality of food consumed with stunting (reduced linear growth), EED, and acute malnutrition	Secondary analysis of an RCT on fortified flours:355 children aged 6–36 mo nested within an RCT conducted in 2 locations in rural Malawi	Multiple linear regression	• Linear growth (LAZ/HAZ)• Acute malnutrition (low MUAC or WHZ)• EED	• Children aged 12–36 mo from the community who had higher consumption of ASF (mostly fish) had improved linear growth.• However, there were no associations between ASF consumption and linear growth among infants 6–12 mo.• No association between ASF consumption and acute malnutrition or EED	ASF consumption supports linear growth in children aged >12 mo in this population, and the most common ASF consumed in this population was fish	Low
Khamis 2019 (24)Tanzania	To examine the association of dietary diversity on nutritional status of children	Cross-sectional study:2960 children aged 6–23 mo	Bivariate and multivariate logistic regression	• Stunting• Wasting• Underweight	• Children who did not consume the minimum dietary diversity were significantly more likely to be stunted and underweight vs. children who did.• There was no association between consumption of the minimum dietary diversity and wasting.• Children who did not consume any meat (including fish) were more likely to be stunted, but no significant difference was found for wasting and underweight between children who did and did not consume meat	Consumption of a diverse diet is associated with a reduction in undernutrition among children 6–23 mo of age in Tanzania. In addition to dietary diversity, ASFs such as meat, fish, milk, and eggs are protective against stunting in children	Medium
Luxwolda 2012 (27)Tanzania	To investigate if lifelong maternal frequency of fish intakes influences maternal DHA during the 3 trimesters of pregnancy and if DHA is passed to the infant at birth and 3 mo postpartum	Cross-sectional study:• 3 tribes with low (0 times per wk), medium (2 times per wk) and high (4 times per wk) fish intake• 205 pregnant women• 63 mother–infant pairs at delivery• 104 different mother–infant pairs 3 mo postpartum	Linear regression	DHA measured in maternal and infant RBC-DHA	• In the tribe with the high-fish diet, maternal DHA at delivery was higher than infant DHA, indicating that DHA was preserved. However, at 3 mo postpartum, the DHA in the infant was higher than the maternal DHA (evidence of biomagnification); these trends were not seen with medium or low fish intake.	High maternal fish intake was associated with high concentrations of DHA during pregnancy. In the high-maternal fish intake group, DHA was preserved in the mother and passed onto the infant at higher concentrations vs. women with medium and low fish intakes	Medium
					• Overall maternal and infant DHA was lower in the medium- and low-fish intake women.• Maternal (but not infant) RBC-AA was higher in the tribe with low fish intake		
Luxwolda 2013 (28)Tanzania	To investigate the association of the frequency of fish intake (indicated by RBC-DHA) with vitamin D concentrations	Cross-sectional study:367 women (138 pregnant and 108 lactating) in 5 ethnic groups with varying fish intakes	Multiple linear regression	Serum vitamin D concentrations	Fish intake was positively associated with serum vitamin D concentrations	Fish consumption, as measured by RBC-DHA content, was a determinant of vitamin D status in Tanzanian women	High
Luxwolda 2014 (29)Tanzania and The Netherlands	To investigate the relation between the frequency of fish intake (indicated by RBC-DHA) and neurologic development in populational differences in fish intakes	Cross-sectional trial:• 97 infants aged 15 wk in 3 groups: low, intermediate, and high fish intake in Tanzania• 15 infants aged 12 wk: low and intermediate fish intake groups in the Netherlands	Linear regression models	• General movements assessed by motor optimality score and observed movement pattern, as a measure of neurologic development	• Motor optimality score as a measure of general movements quality did not differ among the 4 study groups.• There were positive relationships between observed movement pattern and infant RBC-DHA. In this model, infant age and infant RBC-DHA content explained 16% of the variation in the observed movement pattern scores.• Observed movement pattern score was not related to infant RBC-AA	The positive relationship between fish intake (and RBC-DHA) and the number of observed movement patterns of infants aged 3 mo might reflect the connection of DHA with motor development	High
Mank 2020 (34)Burkina Faso	To identify dietary habits (dietary diversity, food variety score, and dietary pattern) that are associated with the growth development of children	Cross-sectional study:514 children aged 8–59 mo	Regression analysis	• LAZ/HAZ• WHZ	• The maize- and fish-based diet was associated with 11% reduction in wasting and a slight decrease (1%) in stunting.• There was a negative trend in HAZ with an increase in food variety and a positive trend in WHZ for an increase in dietary diversity.• A significant trend for associations with WHZ was observed for only the maize- and fish-based diet in the multiple-adjusted model.• No association between maize- and fish-based dietary pattern score and HAZ in the multiple-adjusted model	Higher dietary diversity and food variety and dietary patterns characterized by maize and fish intake appear to be beneficial for growth development of children aged <5 y in Burkina Faso	Low
Marinda 2018 (30)Zambia	To examine associations with fish consumption amounts/patterns and nutritional status in women of reproductive age and their children	Cross-sectional survey:714 mother–child pairs with children aged 6–59 mo	Poisson regression	• Maternal BMI• LAZ, WHZ, WAZ	• No correlation was found between fish consumption and maternal BMI.• A higher proportion of children who consumed fish had normal weight range.	Fish consumption, particularly small fish (*Kapenta*), was associated with reduced prevalence of stunting in children aged <24 mo	Low
					• Fish consumption was associated with linear growth in children aged 6–23 mo.• The quantity of fish consumed by children was significantly associated with reduced prevalence of stunting for children aged 6–23 mo; however, fish consumption and prevalence of stunting were positively associated in children 24–59 mo		
Marques 2008 (53)Brazil	To assess the frequency of maternal fish intake and the association with children's weight and height during the first 5 y	Prospective cohort study:82 mother–child pairs living in urban Brazil	Pearson correlation	At birth, 6 mo, 36 mo, and 60 mo:• WAZ• WHZ• LAZ	Maternal fish consumption frequency had no association with children's growth at the specified ages	Growth of the children was maintained even in women whose diets were transitioning away from fish; thus, a healthy diet appears to be maintained in this population as diets transition	High
Marques 2020 (52)Brazil	To assess intestinal parasitic infections, nutritional status, and anemia status in preschool children living in traditional communities (higher fish consuming) vs. immigrant tin-mining settlement communities in the Amazon basin	Cross-sectional study:247 children in traditional families and 688 in tin-mining familiesOutcomes were measured in preschool children aged 1–59 mo	Spearman correlation within groupsMann–Whitney *U* test to test differences between communities	• Hemoglobin• Fish consumption in children (measured by mercury in hair)• WAZ• WHZ• HAZ• Intestinal parasites	• Fish consumption was higher in the children living in the traditional setting than in the tin-mining communities.• Children in the traditional setting were less likely to be underweight (low WHZ) but more likely to be stunted (low HAZ) vs. the tin-mining community	Linear growth status for children in traditional communities was slightly poorer than for children in tin-mining communities, even though fish consumption was higher. High rates of parasitic infections are an underlying issue for	High
					• The children from the tin-mining group had a higher hemoglobin concentration than the children from the traditional group.• There was a correlation between fish consumption and hemoglobin in the traditional community but not in the tin-mining community	children living in the Amazon basin, which can have a debilitating impact on nutritional status	
Melaku 2018 (20)Ethiopia	To investigate associations between household, maternal, and child dietary patterns and stunting	Cross-sectional study:3788 mother–child pairs with children <5 y	Multilevel linear and Poisson regression	HAZ	• No association between household “fish, meat, and miscellaneous” dietary pattern and stunting or HAZ.• A household and child dietary pattern of higher “dairy, veg, and fruit” was associated with an increase in HAZ in children	Dietary patterns consisting of dairy, vegetables, and fruit at the household and child levels were inversely associated with stunting in this population in Ethiopia	Low
Muthayya 2009 (48)India	To assess associations of frequency of fish intake and ω-3 long-chain PUFA intake during pregnancy with low birth weight in South India	Prospective cohort:676 pregnant women in southern India	Multivariate logistic regression	Mother (measured each trimester):• Height• Weight• BMIChild:• Birth weight	• Women who did not consume fish during the third trimester had significantly higher odds for giving birth to an infant with low birth weight vs. women whose intake was above median.	Low fish intake in the third trimester of pregnancy in Indian women was associated with giving birth to a baby with low birth weight	Medium
					• In multivariate analysis, women in the lowest tertile of EPA intake in the third trimester also had significantly higher odds for low birth weight vs. women in the highest tertile		
Nguyen 2018 (49)India	To examine drivers of change (including meat and fish intake) with hemoglobin and anemia among women and children	Secondary analyses of panel data:• 37,165 pregnant women• 760,460 nonpregnant women of reproductive age• 245,346 children (aged 6–59 mo)	Multivariate regression	In 2006 and 2016:• Anemia status• Hemoglobin	• Hemoglobin improvements in children were most strongly explained by nutrition and health interventions; antenatal care was associated with 18% of anemia reductions.• Hemoglobin improvements in pregnant women were most strongly explained by improvements in schooling (24%) and socioeconomic status (17%).• Women's consumption of fish/meat showed a positive association with child hemoglobin: a 1% hemoglobin change among pregnant women and 3% hemoglobin change among children were associated with increased maternal fish/meat consumption	Key drivers of anemia reduction for children were improved health and nutrition interventions and, for pregnant women, improved education and wealth. Fish/meat consumption also led to an increase in hemoglobin	Low
Pinto 2015 (54)Brazil	To test associations between fish consumption amounts and	Prospective cohort of 225 pregnant women	Linear mixed effects models	At 5–13, 20–26, and 30–36 gestational weeks:Fatty acid	Weekly fish intake was associated with positive changes in EPA, DHA, and total	Fish consumption is associated with positive changes in fatty acid	Medium
	serum fatty acid concentrations (EPA, DHA, total PUFAs) during pregnancy			concentrations (EPA, DHA, PUFAs)	PUFAs	concentrations for pregnant women, which may have further health benefits	
Schipani 2002 (41)Thailand	To compare seasonal dietary intake and nutritional status among children in mixed gardening (fish, vegetables, and small animals) and nongardening families	Cross-sectional study:• 30 gardening and 30 nongardening households• Children aged 1–7 y: each group contained 17 males and 13 females	Paired *t* tests (children from intervention households were matched to nonintervention households)	• Serum ferritin• Hemoglobin• Retinol• WAZ• WHZ• HAZ	• Anthropometric measurements were better among children of gardening families, although the differences were not significant.• There were no significant differences in iron, vitamin A, or hemoglobin between the groups	Findings were inconclusive on whether the mixed gardening intervention was associated with improved nutrition or health outcomes	High
Shariff 2016 (42)Malaysia	To identify the relationship between dietary energy density and nutritional and growth status of children	Cross-sectional study:745 urban children aged 1–10 y (178 <3 y old)	Multivariate regression	• WAZ• HAZ• BMI	• Stunting was associated with a higher dietary energy density, with the highest dietary energy density group consuming significantly more servings of meat, fish, and legumes.• No significant association between dietary energy density and underweight, thinness, or overweight	Future studies should confirm the cause-and-effect relationship between higher dietary energy density and stunting	High
Sparling 2020 (50)Bangladesh	To quantify the association of food security, dietary patterns (including frequency of fish	Cross-sectional study:2599 women of reproductive age in rural Bangladesh	Multivariable logistic regression	Depression screening per the Edinburgh Postpartum Depression Scale	• Food insecurity and poor household food consumption were associated with higher odds of depression.	The prevalence of depression was high among this population and linked to poor food and nutrition security	Medium
	consumption), anemia, and BMI with depression among women of reproductive age				• Consumption of dairy, eggs, fish, and vitamin A-rich and vitamin C-rich foods were associated with reduced odds of depression.• Low BMI was associated with depression in women of reproductive age		
Stuetz 2016 (45)Thailand	To assess the association of the frequency of fish paste consumption with the micronutrient status of pregnant women by trimester in a refugee camp	Two sequential cross-sectional studies:• 553 pregnant women in 2004• 515 pregnant women in 2006	Chi-square analysis	• Hemoglobin• Iron• Zinc• Vitamin A• Vitamin E• Thiamin• Folate	• Daily fish paste consumption was positively associated with iron and vitamin A (retinol and β-carotene) concentrations.• Daily fish paste consumption was negatively associated with vitamin E concentrations	Micronutrient-rich staple foods, such as fermented fish paste, may help to improve iron and vitamin A status during pregnancy, but more research is needed on the link between fish paste and vitamin E	Medium
Tichelaar 1999 (22)South Africa	To investigate differences in undernourished children who received 43-g fish and 7.5-g sunflower cooking oil per day vs. matched (age and sex) well-nourished control group that was not supplemented	Longitudinal case–control study:• Children aged 13–60 mo assigned to 1 control arm (*n* = 50) and 1 case arm (*n* = 52), with the latter receiving fish supplementation for 12 mo• Both groups received the same nutritional counseling	Mixed effects regression	At baseline, 6 mo, and 12 mo:• WAZ• WHZ• HAZ• Iron• Zinc• DHA	• The change (improvement) in HAZ was greater in the treatment arm than in the control arm.• The treatment arm had a bigger change in WAZ than the control during the study period (0–6 mo), but the differences were not significant during the second 6-mo period.• Iron and DHA concentrations were higher in the treatment arm after 12 mo vs. the control arm; zinc was lower	Catfish consumption led to improvements in height, weight, iron status, and DHA status of undernourished children but did not contribute to sustained changes in weight during the second 6 mo of intervention vs. healthy controls	High
Thomas 2010 (21)Malawi	To assess the dietary patterns of HIV-infected pregnant women and associations between nutritional status and birth outcomes	Cross-sectional study:577 HIV-infected pregnant Malawian women using 24-h dietary recall	Multivariate linear regression	• Hemoglobin• MUAC• Arm muscle area• Upper arm fat area	• The dietary pattern with the most fish consumption was associated with higher hemoglobin concentrations.• The dietary pattern with high grain intake showed a higher arm muscle area than the other dietary patterns.• The high grain dietary pattern had lower in mean arm fat area vs. the dietary pattern high in fish, meat, and oil.• MUAC did not vary across dietary patterns	The high-fish diet was associated with better hemoglobin concentrations vs. 2 dietary patterns low in fish among HIV-positive women but was also associated with lower arm muscle and higher arm fat.Though the study controlled for socioeconomic status, this body composition likely reflects a higher degree of physical labor in the dietary patterns of the other groups and reduced access to calorie-rich foods	Medium
Vilela 2015 (55)Brazil	To test associations between dietary patterns and anxiety during and after pregnancy	Prospective cohort:207 pregnant women	Longitudinal mixed effects models	At 5–13, 20–26, and 30–36 gestational weeks and 30–45 d postpartum:• Anxiety symptoms	Women with higher adherence to a healthy dietary pattern (vegetables, fruit, fish, and tea) had lower anxiety scores during pregnancy	Women of reproductive age who consumed a more diverse diet, including vegetables, fruit, and fish, had reduced anxiety symptoms during and after pregnancy	Medium
Woon 2019 (46)Malaysia	To determine the prevalence of and factors associated with developing a vitamin D deficiency in pregnant women in Malaysia	Cross-sectional study:535 pregnant women at ≥28-wk gestation	Generalized linear mixed models	Serum vitamin D concentrations	• Women with a higher intake of dietary vitamin D were less likely to have vitamin D deficiency during pregnancy.• Fish and fish products showed the highest contribution to vitamin D intake (35.8%)	High intake of vitamin D, mainly due to fish and seafood intakes, was a protective factor for vitamin D deficiency among pregnant women in Malaysia	High

1 ASF, animal source food; EED, environmental enteric dysfunction; HAZ, height-for-age *z* score; LAZ, length-for-age *z* score; RCT, randomized controlled trial; RUSF, ready-to-use supplementary food; RUTF, ready-to-use therapeutic food; WAZ, weight-for-age *z* score; WHZ, weight-for-height *z* score.

**TABLE 3:** Impact evaluations included in a systematic review exploring the relationship between fish and fish-based products and nutrition and health outcomes in low- and middle-income countries^1^

Author, year, study location	Evaluation design	Intervention	Outcomes measured	Key findings	Conclusions	Risk of bias
Borg 2020 (38)Cambodia	Cluster RCT:Sample of 293 infants aged 6–11 mo randomly assigned to 3 treatment or control groups, with the former receiving a fish- or non-fish-based treatment for 6 mo	Treatments:• Fish-based RUSF in the form of a wafer• CSB (CSB++)• Micronutrient powderComparator: nonsupplemented	• WAZ • WHZ • HAZ • MUAC	• The RUSF group had an increased MUAC vs. the control.• There were no other differences in the measures between the RUSF groups and the other 3 groups	The RUSF provided minimal protection against ponderal growth faltering and was not protective against stunting	Medium
Chipili 2022 (31)Malawi	Parallel-assigned RCT:Sample of 238 children aged 6 moChildren were followed up at age 12 mo	Treatment: dried small fish (*Chisense*) ground into a powder and given to children in a 12-g daily allotmentComparator: control group was given 12-g daily allotment of sorghum powder	• LAZ • WAZ • Stunting	• The children assigned to the fish powder group had improved LAZ and WAZ.• Fish powder reduced the prevalence of stunting in the treatment arm	Fish powder given to families with children aged 6 mo in rural Zambia may be a viable solution for increasing linear growth	Low
Ikawati 2020 (39)Indonesia	RCT:Sample of 48 children aged 6–24 mo allocated to treatment and control groups, with the former receiving a fish-based supplementary food for 2 mo	Treatment: foxtail millet–tuna biscuit (RUSF)Comparator: standard non-fish-based biscuit	• WAZ • Zinc status	• There was a significant difference in nutritional status (WAZ) intake between the experimental and control groups.• Zinc status improved in the intervention arm	Millet–tuna biscuit (RUSF) consumption increased the nutritional status and zinc status of Indonesian children aged 6–24 mo with WAZ vs. controls	High
Konyole 2019 (25)Kenya	RCT:Sample of 428 infants aged 6 mo who received 9 monthly rations of treatment and comparator products, with the former receiving a fish-based complementary food product	Treatment:• WinFood Classic comprising germinated amaranth (71%), maize (10.4%), small fish (3%), and edible termites (10%)• WinFood-Lite comprising germinated amaranth (82.5%), maize (10.2%), and multimicronutrient premixComparator: fortified	• Iron status and anemia prevalence• Fat-free mass• Fat mass• Length• Weight• MUAC • Head circumference• Skinfolds	• No differences in body composition were observed among the 3 intervention groups over the 9-mo intervention; iron status deteriorated in all treatment groups.• There was a slight increase in anemia prevalence in the WinFood Classic intervention group (73%) vs. the WinFood-Lite and CSB+	The WinFood products did not differ from the CSB in fat-free mass, length gain, and weight gain among infants, but iron status deteriorated significantly with all intervention groups and more so in the group with the nonfortified WinFood Classic product containing ASFs.Adding ASF to a nonfortified	Low
		CSB+		groups (64% and 63%)	complementary food supplement may not be sufficient to improve growth	
Lartey 1999 (36)Ghana	Longitudinal randomized trial:Sample of 190 breastfed infants aged 6–12 mo randomly assigned to receive 1 of 4 foods for 6 mo.Cross-sectional control group of 464 infants recruited between 6 and 12 mo of age who received no intervention for growth comparisons	Treatments: Weanimix with fish powder and Koko (fermented maize porridge) plus fish powderComparators: Weanimix, maize, soybeans, and groundnuts and Weanimix plus minerals and vitamins	At 6 mo (baseline) and 12 mo of age:• Iron• Zinc• Vitamin A• Riboflavin status• Dietary intake• Monthly anthropometric data: weight, length, triceps and subscapular skinfold thickness, MUAC, and head circumference• Morbidity data• The cross-sectional study took a onetime measurement of weight, length, triceps skinfold thickness, MUAC, and head circumference	• Children in the intervention arms had higher WAZ and LAZ than children who received no intervention.• No significant differences between intervention groups in weight or length gain or in iron, zinc, or riboflavin status between 6 and 12 mo of age.• No difference in morbidity outcomes in any of the groups• Infants in the group receiving Weanimix plus minerals and vitamins had higher concentrations of iron and vitamin A status at 12 mo than the other intervention groups	Fortified foods with fish powder may contribute to overall improved growth but was not solely responsible for improved growth	Low
Lin 2008 (26)Malawi	Prospective randomized clinical effectiveness trial:Sample of 216 children enrolled at age 6 mo and randomized to receive fortified spread- or fish powder-based complementary food for 12 mo	Treatment: peanut/soy-based fortified spreadComparator: Corn porridge fortified with fish powderNote: the hypothesis was that the porridge fortified with fish powder would not perform as well on the	From 6 to 12 mo and 12 to 18 mo:• Rates of weight gain• Length gain• Zinc status• Selenium status• Morbidity outcomes	• Children who received fortified spread gained 110 g more from 6 to 12 mo of age than children receiving fish powder, but height from 6 to 12 mo did not differ.• Weight gain did not differ between children receiving either	Fortified spread consumption was associated with better weight gain than the fish powder group from 6 to 12 mo of age. Compared with a peanut/soy-fortified spread, foods fortified with fish powder may not promote as much	Low
	Both complementary foods provided 836 kJ/d from 6 to 9 mo and 1254 kJ/d from 9 to 18 mo	anthropometric outcomes		complementary food product between 12 and 18 mo of age, nor did height from 12 to 18 mo.• Neither complementary food product was associated with significantly improved zinc status. The incidence of fever, cough, and diarrhea did not differ between the groups	growth in infants and young children	
Nurhasan 2018 (40)Cambodia	Single-blind RCT:Sample of 358 infants aged 6 mo enrolled for 9 mo to compare 2 WinFood and 2 CSB+ products, with the former product containing small fish. Food distribution and measurements took place monthly	Treatments: WinFood (small fish and edible spiders) and WinFood-Lite (small fish)Comparators: CSB+ (100% plant based) and CSB+ with 8% dried skim milk powder	ω-3 Fatty acids	• No difference in ω-3 fatty acids between intervention groups.• Prolonged breastfeeding may have a greater impact on blood fatty acids than ω-3 fatty acid intake from complementary foods	Fish consumption in populations where there are high levels of prolonged breastfeeding may not affect ω-3 fatty acid concentrations as much as in populations with lower levels of breastfeeding. However, fish in complementary foods can contribute to dietary diversity and have other benefits after breastfeeding has ceased	Low
Sigh 2018 (57)Cambodia	Single-blind RCT:Sample of 75 children aged 6–59 mo randomly allocated to standard treatment (BP-100) or intervention RUTF (NumTrey) for 2 mo during the home treatment of uncomplicated severe acute malnutrition	Treatment: locally produced RUTF made from rice, soybean, mung bean, canola oil, and small indigenous fish (NumTrey)Comparator: milk-based RUTF (BP-100)	• WHZ • HAZ • MUAC	No differences in weight gain, WHZ, HAZ, and MUAC between the RUTFs	NumTrey is a plausible alternative to BP-100 for treating acute malnutrition in a home setting	Medium
Skau 2015 (44)Cambodia	Single-blind RCT:Sample of 358 infants aged 6 mo enrolled for 9 mo to compare 2 WinFood and 2 CSB products, with the former containing small fish. Food distribution and measurements took place monthly	Treatments: WinFood (small fish and edible spiders) and WinFood-Lite (small fish) + a vitamin and mineral premixComparators: CSB+ (100% plant based) and CSB+ with 8% dried skim milk powder	• Fat-free mass• Iron status• Knee–heel length• MUAC • WAZ • LAZ	• No differences in fat-free mass or iron status were observed between any of the products; iron status deteriorated in all groups.• No differences were found in WAZ or LAZ	Small indigenous fish may be a good alternative to milk when considering locally made products for treating malnutrition	Low
Thacher 2015 (35)Nigeria	RCT:Sample of 88 Nigerian children aged 6–15 mo with active rickets who received 1 of 2 treatments (1 fish based) daily for 24 wk	Treatment: ground catfish (952-mg elemental calcium)Comparator: powdered limestone (920-mg elemental calcium)	• BMD (radiographic healing)• Vitamin D• Calcium status	Both groups had improvements in BMD, vitamin D, and calcium status	Treatment with calcium as ground catfish or limestone is viable to promote healing among Nigerian children with rickets and to improve calcium and vitamin D status.Indigenous food sources, such as ground dried catfish, can replace more expensive calcium supplements	Low

1 ASF, animal source food; CSB, corn–soy blend; HAZ, height-for-age *z* score; LAZ, length-for-age *z* score; RCT, randomized controlled trial; RUSF, ready-to-use supplementary food; RUTF, ready-to-use therapeutic food; WAZ, weight-for-age *z* score; WHZ, weight-for-height *z* score.

### Interventions used in impact evaluations

Of the impact evaluations in which fish was given in the complementary feeding period, 1 study provided fish powder alone (31), 1 used fish added to a corn porridge (26), 1 used a Weanimix formulation that included fish (36), and 2 used WinFood formulations with fish (25, 44). The WinFood studies—1 in Cambodia and 1 in Kenya—compared WinFood with fish against corn–soy blends (CSBs). In Cambodia, researchers used 2 formulations of WinFood: both formulations started with a base of traditional rice porridge (*borbor*) and then incorporated either fish and edible spider or fish only, with a vitamin–mineral mix to the fish-only formulation (44). In Kenya, 1 WinFood treatment had a base of germinated amaranth and maize with fish and edible termite, and 1 formulation had a micronutrient premix instead of fish and termite (25).

Ready-to-use supplementary foods (RUSFs) are complementary foods designed to prevent malnutrition, whereas ready-to-use therapeutic foods (RUTFs) are designed to treat malnutrition (59). The randomized controlled trial that evaluated an RUSF intervention used an RUSF made of soy, mung bean, coconut, multiple-micronutrient premix, icing sugar, maltodextrin, and canola oil mixed with the fish paste, which was piped into a wafer made from rice flour, egg, water, sugar, salt, and coconut with a small amount of vanilla or sesame seed (44). A study conducted in Indonesia evaluated the use of an RUSF cookie, with foxtail millet and tuna listed as the only ingredients (39).

In the trials that examined the impact of fish-based products on malnourished children, 1 trial investigated use of an RUTF that consisted of rice, soybean, mung bean, canola oil, and small indigenous fish packed into a wafer (57), in a similar formulation to the RUSF developed by the same research group (44). In a study that recruited children in Nigeria with rickets (a bone malformation often due to prolonged inadequate vitamin D or calcium intakes), ground catfish meal was compared with powdered limestone as a source of calcium in the children's diets over a 24-wk period (35).

Compliance measures included visual approximation (26), weight (35, 40), count (44, 57, 31), and self-reporting (25, 36, 38) of complementary food consumed. One study did not report any compliance measures (39).

### Fish exposures in observational studies

We grouped fish exposures into direct or indirect fish consumption (Table 4). Exposures were considered direct fish consumption in surveys that recorded fish or aquatic food consumption in mothers (27–30, 32, 48, 50, 51, 53, 54) or infants/children (22, 23, 30, 47, 52, 56, 31) without other foods. Exposures were defined as indirect fish consumption when a composite measure was used with other foods (e.g., meat, vegetables). Two studies (18, 41) that assessed the effects of living in fish-farming households were also considered under the indirect fish consumption category. Composite measures included ASF consumption (19, 24, 49, 56), dietary patterns (20, 21, 34, 42, 55), iron-rich food consumption (32), ω-3 fatty acid-rich food consumption (37), and vitamin D-rich food consumption (46).

**TABLE 4:** Evidence gap map of fish consumption and nutrition and health outcomes in the first 1000 d of life from studies included in a systematic review exploring the relationship between fish and fish-based products and nutrition and health outcomes in low- and middle-income countries^1^

	Maternal outcomes					Infant outcomes			
Exposure: intervention	Anthropometric	Fatty acids	Micronutrients	Other health	Neonatal outcomes	Anthropometric	Fatty acids	Micronutrients	Other health
Direct fish consumption									
Complementary food and ready-to-use-supplementary food with fish						Borg 2020 (38)^M^ Konyole 2019 (25)^L^Skau 2015 (44)^L^Lin 2008 (26) ^L^Lartey 1999 (36) ^L^Ikawati 2020 (39)^H^	Nurhasan 2018 (40)^L^	Konyole 2019 (25)^L^Skau 2015 (44)^L^Lin 2008 (26) ^L^Lartey 1999 (36)^L^Ikawati 2020 (39)^H^	Lin 2008 (26)^L^Lartey 1999 (36)^L^
Ready-to-use-therapeutic food with fish or fish given to treat malnutrition						Sigh 2018 (43)^M^Tichelaar 1999 (22)^M^		Tichelaar 1999 (22)^M^	Thacher 2015 (35)^L^
Infant and child fish consumption						Marques 2020 (52)^H^Kaimila 2019 (23)^L^Marinda 2018 (30)^L^Chipili 2022 (31)^L^Headey 2017 (56)^L^		Marques 2020 (52)^H^Hawlader 2019 (47)^M^	
Maternal fish consumption	Marinda 2018 (30)^L^	Pinto 2015 (54)^M^Luxwolda 2012 (27)^M^	Luxwolda 2013 (28)^H^Stuetz 2016 (45)^M^Anlaaku 2017 (32)^M^	Sparling 2019 (50)^M^	Muthayya 2009 (48)^M^Marques 2008 (53)^H^	Cunha 2018 (51)^M^Marques 2008 (53)^H^	Luxwolda 2012 (27)^M^		Luxwolda 2014 (29)^H^
Indirect fish consumption									
Animal source food consumption (including fish): maternal and child	Bosha 2019 (19)^M^		Nguyen 2018 (49)^L^			Khamis 2019 (24)^M^		Nguyen 2018 (49)^L^	Kaimila 2019 (23)^L^
Dietary pattern (including fish): maternal and child	Thomas 2010 (21)^M^		Woon 2019 (46)^M^Thomas 2010 (21)^M^	Vilela 2015 (55)^M^	Angkasa 2017 (37)^M^	Mank 2020 (34)^L^Melaku 2018 (20)^L^Shariff 2016 (42)^H^			Dei-Tutu 2020 (33)^M^
Residing in fish-farming household						Aiga 2009 (18)^L^Schipani 2002 (41)^H^		Schipani 2002 (41)^H^	
Total per outcome^2^	3	2	6	2	3	21	2	10	6

1 The risk of bias for each study is indicated in superscript parentheses: H, high risk of bias; L, low risk of bias; M, medium risk of bias. Risk of bias was determined per the following critical appraisal tools specific to each study design: revised Cochrane risk-of-bias tool for randomized trials (13), ROBINS-I (Risk of Bias in Non-Randomized Studies of Interventions) (14), Critical Appraisal Skills Program cohort (15) and case–control (16) checklists, and Appraisal Tool for Cross-Sectional Studies (17).

2 Totals show the number of studies per outcome, with higher numbers indicating more studies assessing each outcome.

The most frequent exposure assessed in observational studies was maternal fish consumption (*n* = 9). Exposure data collection methods included quantitative and qualitative 24-h dietary recalls (19–21, 23, 24, 30, 36, 42, 50, 56), FFQs (34, 37, 46, 48, 49, 54, 55), nonvalidated food questionnaires (27, 29, 45, 47), and nonvalidated but pretested questionnaires (32). The weakest fish consumption measures were nonvalidated proxy measures such as hair mercury concentrations (51, 53) and RBC ω-3 fatty acid concentrations (28).

### Outcome variables

Outcome variables were highly heterogeneous, falling under maternal, neonatal, and infant outcomes, with maternal and infant outcomes categorized into anthropometric, fatty acid, micronutrient, and other health and/or morbidity outcomes (Table 4). Three studies assessed maternal anthropometric outcomes, with indicators covering BMI (19, 30) and MUAC (19, 21), including arm fat and muscle area (21). Two studies analyzed maternal fatty acid indicators (27, 54). A range of maternal micronutrient indicators was cited, such as anemia prevalence (32, 49), iron (21, 45, 49), zinc (45), vitamin D (28, 46), vitamin E (45), and vitamin A (45). Two studies assessed maternal depression and anxiety—one postpartum (50) and the other during pregnancy (55). Three studies reported on neonatal outcomes: low birth weight (37, 48, 53), birth length (37, 53), and head circumference (37).

Most articles assessed infant and child outcomes, with the most reported being anthropometric measures. Growth indices included length- or height-for-age *z* score (LAZ or HAZ) (22, 23, 30, 34, 36, 38, 41, 42, 44, 51–53, 57, 31), weight-for-age *z* score (WAZ) (22, 30, 36, 38, 39, 41, 42, 44, 51–53, 57, 31), and weight-for-height *z* score (WHZ) (22, 30, 34, 36, 38, 41, 42, 44, 51–53, 57). Anthropometric indices consisted of MUAC (36, 38, 44, 57), length and weight (25, 26), head circumference (25, 36, 44), and skinfold thickness (25, 36, 44). Six studies examined population-level indicators such as stunting (18, 20, 24, 30, 42, 56, 31), wasting (18, 24), underweight (18, 24, 42), acute malnutrition (23), and overweight/obesity prevalence (42). Only 2 studies examined body composition outcomes: fat-free mass (25, 44) and fat mass (25). Four articles examined fatty acid concentrations (22, 27, 29, 40). Several studies reported iron status (36, 41, 44) and iron-related indicators such as ferritin (22, 25, 36, 41, 44), transferrin receptors (25, 36, 44), and hemoglobin concentrations and/or anemia (25, 36, 41, 49, 52). Vitamin A (36, 41), vitamin D (22, 47), selenium (26), and zinc status (22, 26, 36, 39) were less commonly examined. Some studies examined other health and morbidity outcomes in infants, such as TSH concentrations (33), motor development (29), rickets (35), and environmental enteric dysfunction (23), as well as fever, cough, and diarrhea (26).

### Evidence from impact evaluations

Of the trials that assessed the impact of complementary food, RUSF, or RUTF with fish added on infant and child outcomes, all were randomized controlled trials (*n* = 9) except for Tichelaar et al. (22) (Table 4). Thus, the Tichelaar et al. study, which is longitudinal, is summarized in the observational studies section. We uncovered 1 impact evaluation that directly measured fish consumption in the form of a 12-g “daily dose” of fish powder given directly to families (31). This 2022 study is included in our review as it was discovered as a planned clinical trial in our initial search (58) and results from the trial were published during the revision of this article.

#### Anthropometric outcomes

Among the 6 studies that measured fish as an intervention to prevent linear growth faltering (stunting), Skau et al. (44) hypothesized and found that the fish-based intervention worked as well as a milk-fortified treatment group, and 3 other studies hypothesized that the fish intervention arm would perform better than a control group (25, 36, 31). Only Chipili et al. (31) noted that fish powder significantly improved LAZ. Borg et al. (38) found no difference in linear growth between the fish-based intervention and an unsupplemented control group. Lin et al. (26) hypothesized that the fish intervention (corn porridge fortified with fish powder) would perform less well than the comparator of a peanut/soy-fortified spread; however, the authors did not observe differences in linear growth between the groups of infants.

Our review uncovered 1 trial investigating a fish-based RUTF on the treatment of severe acute malnutrition (57). The trial was designed to evaluate the equivalency of fish-based RUTF (NumTrey) to the standard-of-care RUTF (BP-100 biscuit) among children diagnosed with uncomplicated severe acute malnutrition in Cambodia. The authors did not find any significant differences between the groups in terms of weight gain, WHZ, HAZ, or MUAC; children in both groups improved after 56 d of the intervention. However, the children in the BP-100 group gained more height after 56 d, though they were slightly shorter and smaller at baseline than the children in the NumTrey group (57).

#### Nutrient status and morbidity outcomes

Three trials conducted in Ghana (36), Kenya (25), and Cambodia (44) investigated the role of fish and fish-based products on iron status or anemia. Findings from all trials suggested an overall deterioration in iron status over time, regardless of the intervention. Specifically, in Kenya, Konyole et al. (25) found that, compared with the CSB groups, both WinFood arms of the study had a decrease in hemoglobin, and the WinFood classic group with fish added had a slightly higher anemia prevalence than the CSB group. Similar findings were reported by Skau et al. (44) from a study in Cambodia, in which iron status deteriorated over the intervention period in both WinFood groups (both had ASFs added) and the CSB groups. Research conducted in Ghana by Lartey et al. (36) revealed no statistically significant difference in iron status among the 4 types of complementary food, only 1 of which contained fish, but again iron status deteriorated over the 6 mo.

Six studies measured the status of other minerals and vitamins, such as zinc, riboflavin, selenium, calcium, vitamin A, and vitamin D. We uncovered 3 trials that investigated the impact of fish and fish-based products on zinc status (26, 36, 39). Two did not find any evidence of an improvement in zinc status (26, 36). Yet, a trial conducted in Indonesia found that providing millet biscuit fortified with tuna did improve the zinc status of children aged 6–24 mo (39). Also, we did not find evidence of an improvement in selenium (26), riboflavin, or vitamin A status (36). In a study in Nigeria of children with rickets, 2 treatment groups (ground catfish meal compared with powdered limestone) experienced increases in BMD, calcium, and vitamin D status (35). Nurhasan et al. (40) looked at the same cohort in Cambodia as Skau et al. (44) to investigate serum ω-3 fatty acids in groups given WinFood with small fish added and the CSB groups and did not find any differences.

In addition to assessing nutrition outcomes, 2 trials included morbidity outcomes (prevalence of fever, cough, diarrhea, and respiratory infections) as secondary outcomes (26, 36). Neither trial found significant differences with respect to morbidity outcomes in children fed fish or nonfish foods.

### Evidence from observational studies: Direct fish consumption

#### Infant and child fish consumption

Our review uncovered 1 observational trial (case–control) that provided fish directly to children experiencing malnutrition (22). The trial was done in South Africa and provided families of undernourished children with catfish fillets 3 times per week for 1 y. The weight and length gain of the undernourished children was compared with a control group of well-nourished children who were not given catfish; children in the undernourished group gained height and weight faster than the well-nourished control. The catfish-supplemented group also had a higher concentration of DHA and iron than healthy controls after 12 mo, but zinc status deteriorated among the supplemented group (22).

Direct fish consumption was measured in nonmalnourished infants and young children in 5 observational studies (Table 4), and most provided positive evidence for fish consumption during infancy and early childhood and for nutrition and health outcomes. Out of the 5 studies, 3 examined amount of fish consumed (23, 30, 52) and 2 analyzed the frequency or prevalence of consumption (47, 56). Four studies were cross-sectional (23, 30, 52, 56) and 1 was case–control (47). Results were mixed. Three studies showed positive associations with HAZ in certain age groups (30, 56). In a study in Malawi, children aged 12–36 mo consuming ASFs (mainly made up of fish) had a higher HAZ (23). In Zambia, Marinda et al. (30) revealed a significant correlation between the quantity of fish consumed by infants aged 6–23 mo and stunting but not between infant fish intake and WHZ. However, Marinda et al. noted a negative association between fish consumption and HAZ in children ≥24 mo. In a study of 46 low- and middle-income countries, Headey et al. (56) cited an association between fish consumption and a reduced prevalence of stunting. On the contrary, Marques et al. (52) reported that Brazilian children living in a traditional fishing community had a lower HAZ score when compared with children living in tin-mining communities that consumed less fish. Yet, when Marques et al. investigated associations within groups rather than between, they found that hemoglobin concentrations were correlated to fish consumption only in children in traditional high fish-consuming communities (i.e., they did not find this relationship in the tin-mining community). The case–control study focused on fish consumption in infants and young children and the association with vitamin D status and showed that infants aged 12–24 mo in Bangladesh who did not consume fish were more likely to be vitamin D deficient (47).

#### Maternal fish consumption

Direct fish consumption among mothers, whether measured by frequency or in quantitative amounts, was measured in 11 observational studies, the highest number of all the exposures. However, 3 studies were reported from the same population in the Amazon basin in Brazil (51–53), and another 3 studies were from 1 trial conducted among tribal groups in Tanzania (27–29). All but 2 studies (30, 54) relied on the frequency of fish consumption rather than the quantity.

Findings were mixed on the benefits of maternal fish consumption on maternal outcomes from the 7 studies that investigated this relationship. Multiple studies found an association between maternal fish intake during pregnancy and fatty acid concentrations (27, 54) and vitamin D concentrations (28). Stuetz et al. (45) reported a positive association between daily fish paste consumption and iron and vitamin A status but a negative association with vitamin E among pregnant women in Thailand. Findings from Sparling et al. (50) in Bangladesh showed a correlation between fish consumption and mental health, with peripartum women who ate fish on the previous day having lower odds of depression. In urban Zambia, no correlation was revealed between fish consumption and women's BMI (30). A trial in Ghana cited no association between consuming fish/snail and anemia among pregnant women, after adjusting for confounding factors (32).

From the 5 studies examining maternal fish consumption and its association with neonatal outcomes, findings were again heterogeneous. Muthayya et al. (48) indicated that women in India who did not eat fish during the third trimester had a significantly higher risk of delivering a baby with a low birth weight. Yet, evidence from Marques et al. (53) suggested that neither birth length nor birth weight differed by frequency of fish consumption among women in Brazil, and no association was noted with HAZ, WAZ, or WHZ when the children were followed up for 5 y (53). Cunha et al. (51), researching a similar population, also found that the frequency of fish consumption was not associated with HAZ, WAZ, or WHZ in children at 6, 24, and 59 mo. A study in Tanzania did find that maternal fish consumption led to increased infant fatty acid concentrations at 3 mo postpartum but did not report if this then led to improved cognitive development in those infants (27). A cross-sectional study based on the same data assessed the association of maternal fish consumption on infant motor development and revealed that increased concentrations of DHA led to a significant increase in observed movement patterns (a measure of infant neurodevelopment) in 3-mo-old infants as compared with infants who had lower DHA in their RBCs (29).

### Evidence from observational studies: Indirect fish consumption

#### ASF consumption

Two studies examined the association of ASF consumption (including fish) with maternal outcomes (Table 4). The first was a cross-sectional study in Ethiopia that identified no association between ASF consumption by women and the MUAC or BMI of women of reproductive age (19). The second, a large panel survey, found that weekly consumption of fish or meat by women was associated with a 1% increase in hemoglobin concentrations among pregnant women (49).

Three studies examined the correlation between fish/meat consumption in women or children and infant outcomes (Table 4). Findings by Khamis et al. (24) demonstrated that in Tanzania, children aged 6–23 mo who did not consume any fish/meat had a higher likelihood of becoming stunted. However, in terms of wasting and underweight, no significant difference was seen between children who did and did not consume fish/meat (24). Nguyen et al. (49) found that mothers’ fish/meat consumption accounted for a 3% increase in hemoglobin in their children. With respect to morbidity outcomes, Kaimila et al. (23) in Malawi did not find a significant association between children's ASF consumption and acute malnutrition or environmental enteric dysfunction in Malawi.

#### Dietary patterns

Three studies assessed whether overall dietary patterns (not just ASFs) that included fish were associated with maternal outcomes (Table 4) (21, 46, 55). Thomas (21) analyzed associations with anthropometric indicators among HIV-infected pregnant women in Malawi based on different dietary patterns. Pregnant women who consumed a dietary pattern with high fish, meat, and oil showed no difference in MUAC when compared with women who had nonfish dietary patterns. Yet, pregnant women who had the fish-based dietary pattern had significantly lower mean arm muscle area and higher arm fat area than those who consumed a high-grain dietary pattern. Findings from Thomas also indicated that pregnant women who had a dietary pattern based on high fish, meat, and oil had higher hemoglobin concentrations than those with nonfish dietary patterns. A study in Malaysia demonstrated that a dietary pattern that included fish was protective against pregnant women developing a vitamin D deficiency (46). Fish-based dietary patterns among pregnant women may be linked to mental health outcomes. In a study by Vilela et al. (55), pregnant women in Brazil who consumed a dietary pattern based on fish, vegetable, fruit, and tea had lower anxiety during pregnancy as compared with a dietary pattern based on rice, bean, meat, and egg or bread, sugar, fat, fast food, and snack.

In a cross-sectional study, Angkasa et al. (37) assessed the relationship between maternal dietary intake of ω-3 fatty acids (including but not limited to fish) and neonatal outcomes in Indonesia and reported varying results for birth weight based on the type of fatty acid. Although maternal ALA intake was significantly associated with birth weight, DHA and EPA intakes were not. No significant associations were reported for the relationship between intakes of ω-3 fatty acids and birth length/head circumference (37).

Four cross-sectional studies (20, 33, 34, 42) evaluated the impact of either mother or child dietary patterns (including but not limited to fish) on infant/child outcomes (Table 4). No link was identified between maternal seafood consumption and infant TSH concentrations (33). In Burkina Faso, Mank et al. (34) reported a significant correlation between fish- and maize-based dietary patterns in infants aged 8–59 mo on WHZ but not HAZ. Likewise, Melaku et al. (20) reported no significant association between stunting in infants/children aged 6–59 mo and a household, maternal, or child dietary pattern of fish and meat. Curiously, findings from Shariff et al. (42) in Malaysia suggested that higher consumption of fish, meat, and legumes among children 1–10 y old was linked with higher rates of stunting; however, the researchers did not find this link with underweight, thinness, or overweight/obesity.

#### Residing in fish-farming households

Two cross-sectional studies (18, 41) analyzed the impact of residing in fish-farming households on nutrition outcomes (Table 4). Although findings from Aiga et al. (18) in Malawi showed a significant difference in the prevalence of underweight in children between fish-farming and non–fish-farming households, findings from Schipani et al. (41) in Thailand suggested no significant differences in *z* scores or micronutrient status (hemoglobin, iron, and vitamin A concentrations) in infants/children.

### Risk of bias

A summary of the risk of bias of each study is presented in the online **Supplemental Results**. The ranking of each study (low, medium, and high risk of bias) is presented in Table 2 for the observational trials and Table 3 for the impact evaluations.

## Discussion

**FIGURE 2:**
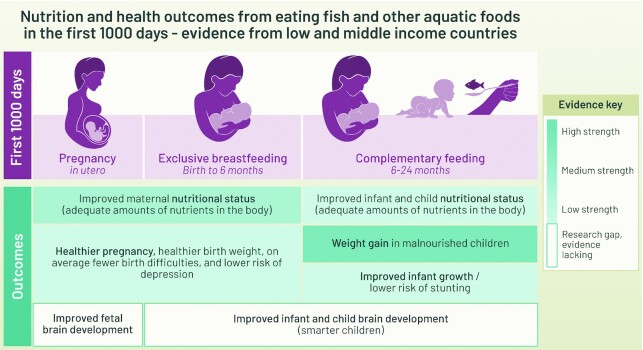
Research outcomes ranked by the strength of their evidence in the systematic review investigating the relationship between fish and fish-based products and health and nutrition outcomes in low- and middle-income countries. Available evidence and the strength of the evidence were considered. The strength of evidence was determined by critical appraisals conducted by 2 independent researchers using predefined rubrics specific to the study design, while considering the robustness of the study design.

Our systematic review investigating the relationship between fish and fish-based product consumption and nutrition/health outcomes revealed a sparse evidence base, showing a slightly positive but still inconclusive association. Reviews of other ASFs have been unable to draw conclusions given the heterogeneity of results (9, 10, 60). Overall, we found *1*) strong evidence that fish intake improves weight gain in malnourished children, *2*) medium-strength evidence that fish intake improves maternal nutrient status, and *3*) emerging evidence that fish intake improves child growth (Figure 2).

We did not find any studies that measured child development as an outcome in any time point in the first 1000 d, highlighting an important research gap.

We uncovered 18 observational studies in pregnant and lactating women, 12 of which found a positive relationship with at least 1 maternal, infant, or child nutrition or health outcome. We also uncovered 10 impact evaluations, all of which focused on outcomes in infants and children. Among all the types of studies done in infants and children, 15 of 23 showed a positive relationship between fish consumption and at least 1 nutrition or health outcome. Most observational studies were cross-sectional, so in many cases, the direction of causality is unclear. Increasing fish consumption in low- and middle-income countries holds promise for improving nutrition and health outcomes, but more robust research is needed.

In accordance with other reviews of ASFs (10) and nutrition-sensitive agriculture programs (61), we found little evidence that interventions using fish or fish-based products had an impact on stunting. However, in trials conducted in 46 low- and middle-income countries and Zambia, Headey et al. (56) and Marinda et al. (30) respectively showed a positive association between fish consumption and linear growth in children 6–24 mo old. In addition, a trial that published results in 2020 indicated that the daily provision of 12 g of fish powder improved LAZ (31). Marinda et al. also demonstrated the importance of disaggregating children by age group to closely examine when fortification of diet with ASF is likely to have the greatest impact, given that the positive association between fish consumption and linear growth was observed in children aged 6–23 mo and not 24–59 mo. These findings emphasize the importance of intervening at the start of the complementary feeding period for improved child growth.

Stunting, as measured by LAZ or HAZ (for those <2 y and >2 y, respectively), is a difficult indicator on which to detect improvements, often requiring large sample sizes in addition to a robust trial design. Given the resource-intensive nature of randomized trials, we speculate that many impact evaluations in this review were underpowered. Sample sizes in the impact evaluations ranged from ∼25 to 100 children per treatment arm, but in general, larger sample sizes are needed to detect an effect on linear growth in children (62). Nutrition-sensitive agriculture interventions often are based around a theory of change that concludes with improved nutrition and health outcomes, but linear growth is a complex biological phenomenon that has proven difficult to investigate (61). Furthermore, stunting is not always the most appropriate indicator to measure in an intervention, and nutrition-sensitive interventions are better suited to measure impacts on diet and household income (63).

A similar lack of effect was uncovered for impact evaluations investigating micronutrient status and anemia in children. None of the fish-based interventions improved iron or hemoglobin. In fact, all the children in the studies experienced a decline in iron status over time, which may be due to nondietary factors such as chronic inflammation resulting in poor iron absorption (64) or untreated malaria infections (65). Additionally, genetics (66) and malaria infections (67) play a role in iron status, yet these factors were not measured in any of the trials in our review. One study in Indonesia did see an improvement in zinc status by giving children a tuna-fortified biscuit, but this study was classified as having a high risk of bias (39).

Additionally, none of the studies on infants and children measured cognitive outcomes, which is an important gap to note, considering that fish are generally a good source of nutrients, such as ω-3 fatty acids, which are important for cognitive development (5, 68, 69). Maternal seafood consumption has been shown to be associated with neurocognitive outcomes in children, but this finding has yet to be replicated in low- and middle-income countries (8). There are several trials showing that fish consumption improves the ω-3 fatty acid content of breastmilk (68–70), but it is unclear whether maternal fish consumption increases the ω-3 fatty acid concentration enough to influence cognitive development. Because children in low- and middle-income countries face many challenges that negatively influence cognitive development, it is urgent to investigate all resources that could mitigate these challenges (71).

There were only 2 impact evaluations: one in Nigeria with catfish meal (35) and another in Cambodia with an RUTF made with a small indigenous fish species that included malnourished children (57). The study done in Nigeria demonstrated that catfish meal provides a feasible solution for addressing calcium and vitamin D deficiencies in malnourished children (35). In Cambodia, using a small indigenous fish in an RUSF was as effective in promoting child growth among malnourished children as a milk-based RUSF (57). This is promising given that small indigenous fish species are a more acceptable and locally available ASF than milk in certain parts of Southeast Asia (43). A longitudinal study that provided catfish fillet to families of malnourished children in South Africa showed an improvement in growth (22). However, the children were compared with well-nourished children; therefore, it is unclear if the improvements seen were a result of the catfish fillet improving growth or the children were already headed toward recovery from a malnourished state.

When evaluating the lack of impact of fish and fish-based interventions on nutrition outcomes, one must consider the food matrix within which the fish is delivered. Many fish-based interventions mixed fish powder with a high-phytate food, such as maize. Phytates bind to iron and zinc and reduce the availability of these minerals to be absorbed in the gastrointestinal tract (72). Although fish is said to have an “enhancing factor” that increases the bioavailability of iron from other foods consumed with the fish (73), it is not known if this factor can overcome the antinutrient activity of a high-phytate meal. Although fish powder is considered nutrient dense, interventions in which fish powder was mixed with high-phytate foods likely dampened the effect on micronutrient uptake (5). Additionally, the amount of fish used in the interventions might have been too small to affect health and nutrition outcomes, especially with an outcome such as child growth, which is influenced by many factors, including chronic infections (74). Indeed, a recent review of ASFs on child growth excluded many fish interventions from the analysis because the amount of fish added to the complementary food in the intervention did not meet the minimum threshold for inclusion (9). This highlights the importance of ensuring that the portion sizes of ASF-based interventions, including fish, are given in sufficient amounts to meet the recommended nutrient intakes for the target population.

The evidence for maternal consumption of fish and the correlation with improved status of maternal hemoglobin, ω-3 fatty acids, iron, and vitamin D are encouraging and support the recommendation that fish consumption in pregnant women should be prioritized, supporting findings from other studies in high–fish-consuming communities in high-income countries (75). However, aside from Marinda et al. (30), all the studies on maternal fish consumption were medium to high risk of bias. Additionally, most of these studies relied on the frequency of fish consumption rather than the quantity consumed. More robust studies are needed that measure the precise amounts of fish consumed to detect an association with maternal and child outcomes.

Fish provided to families must be safe, and although food safety was outside the scope of this review, contaminants are a concern, as with all perishable foods. Contaminants are often high in fish, and the mercury concentration is so high in fish in the Amazon basin that mercury was used as a marker of fish consumption in the studies conducted in the Latin American region (51, 53). However, a study from the Seychelles that was not in our review reported no adverse association between maternal mercury intake from fish and neurocognitive outcomes in children, and it found that high maternal DHA intake remained important, even if the fish contained mercury (76). In other regions, fish drying and smoking may lead to harmful contaminants such as aflatoxin (77) and polycyclic aromatic hydrocarbons (78). Thus, food safety remains an important consideration when developing fish-based interventions and programming, and supply chain interventions to improve fish products are needed.

### Limitations

Limitations of this study include that it was not preregistered on any platform; regardless, the only change that we made in the methods over the course of the study was to expand the search into the year 2020. Additionally, we included articles written only in English, which may bias the results toward English-speaking countries.

### Conclusion

Our findings add to the global discourse that food- and agriculture-based interventions should go beyond targeting 1 outcome (i.e., stunting or anemia reduction) and focus on improving dietary quality through sustainable, nutrition-sensitive food systems (61). This is so for interventions targeting the critical 1000-d window, as well as interventions targeting households and people outside the 1000-d window more broadly. Although attention is shifting to improving food systems overall, much of the robust research done on the first 1000 d has been on manufactured products—for example, an LNS (lipid-based nutrient supplement) and a micronutrient powder that are shelf stable and designed to provide nutrients in amounts that children aged 6–24 mo need. These products have been justifiably extensively researched through robust randomized trials as well as meta-analyses (79–81). Most recently, a meta-analysis based on individual data (rather than study-level data) was done to examine the impact of LNS on health and nutrition outcomes, with favorable results (82). However, solutions that are more likely to be available in local settings, such as fish and other ASFs, have not been well researched, and this needs urgent redressing. These solutions are especially relevant for rural settings, given that solutions such as large-scale food fortification sometimes do not meet the nutrient requirements of the rural poor, as it does not often purchase or consume enough of the fortified foods (83). There has been some robust research on how the provision of ASF can improve outcomes in the first 1000 d, such as evaluations of programs providing livestock (84) or eggs to families (85, 86), but more is needed.

Regardless, fish and the broader category of aquatic foods are gaining recognition in global discourse. An article under the Blue Foods Assessment project was recently released that published the nutritional values of >3000 aquatic food species and outlined their importance to food and nutrition security (87). The current enthusiasm for the potential of fish and other aquatic foods to address malnutrition adds to the urgency of the need for more robust studies. Future studies can design interventions by using locally available foods (e.g., dried small fish) provided to pregnant and lactating women and children aged 6–23 mo and by measuring cognitive development outcomes as well as nutrition outcomes. These studies can also be extended outside the 1000-d window to school-aged children, when the impacts of improved cognitive development are more likely to be detected. In designing new studies, attention should be paid to the role of phytates, which limit iron and zinc absorption. Furthermore, studies of homestead food production with small fish through integrated aquaculture have not been rigorously evaluated for their impact on nutrition and health outcomes, despite analyses providing evidence that interventions such as these are more cost-effective than other vitamin A programs (88).

The environmental and nutritional characteristics of fish and other aquatic foods mean that they have a high potential for addressing the burdens of malnutrition in the first 1000 d, but to date this has not been demonstrated clearly in the scientific research. Food systems that leverage aquatic foods have the potential to improve dietary quality with a relatively low environmental impact, and policy makers need much clearer evidence from researchers on the potential benefits of using these foods to address malnutrition. More attention is needed in this area so that stakeholders can have a clear idea of who will benefit and how much, when investing in fish and fish-based products to address malnutrition and improve the nutrition and health of mothers and children in developing countries.

## Supplementary Material

nmac102_Supplemental_File

## Notes

This publication is funded as part of the CGIAR Research Initiative on Resilient Aquatic Food Systems for Healthy People and Planet. We thank all funders who support this research through their contributions to the CGIAR Trust Fund. LO was supported by Research England through the Food and Nutrition Security Initiative at the University of Greenwich.

Author disclosures: The authors report no conflicts of interest.

Supplemental Results are available from the “Supplementary data” link in the online posting of the article and from the same link in the online table of contents at https://academic.oup.com/advances/.

KAB and JS contributed equally to this work.

Abbreviations used: ASF, animal source food; CASP, Critical Appraisal Skills Program; CSB, corn–soy blend; HAZ, height-for-age *z* score; LAZ, length-for-age *z* score; ROBINS-I, Risk of Bias in Non-Randomized Studies of Interventions; RUSF, ready-to-use supplementary food; RUTF, ready-to-use therapeutic food; WAZ, weight-for-age *z* score; WHZ, weight-for-height *z* score.
