# Supplementary material for: Fish and Fish-Based Products for Nutrition and Health in the First 1000 Days: A Systematic Review of the Evidence from Low and Middle-Income Countries
Source: Adv Nutr. 2022 Sep 27;13(6):2458–87. doi: 10.1093/advances/nmac102 (PMC9776644; doi:10.1093/advances/nmac102)
Supplement: nmac102_Supplemental_File [file nmac102_supplemental_file.docx]

**Supplementary Results for: Fish and Fish-based Products for Nutrition and Health in the First 1000 Days: A Systematic Review of the Evidence from Low- and Middle-income Countries – Byrd et al**

Risk of bias

Of all 39 studies included in this systematic review, 25 studies were found to be at medium to high risk of bias.

*Risk of Bias (ROB) in studies investigating complementary foods and RUSF/RUTF*

All but two trials on complementary foods/RUSF with fish were rated as low risk of bias. Borg et al (37) was rated as medium risk of bias due to inadequate adjustment and reporting on large loss to follow up. Ikawati et al (38) was rated as having a high risk of bias because of inadequate reporting on recruitment, randomization, blinding, outcome measurement, and statistical analysis methods.

Two trials (22,56) on RUTF were rated as medium risk of bias due to inadequate adjustment and reporting on large loss to follow up. Thacher et al (34) was rated as having a low risk of bias.

*Risk of Bias (RoB) in studies investigating direct fish consumption*

The randomized controlled trial by Chipili et al, looking at fish powder consumption was ranked as low risk of bias (93). The five longitudinal studies (46,47,50,52,53) (Table 4) on direct fish consumption were all rated between medium and high risk of bias, with the primary reasons for medium risk of bias being inappropriate exposure measurements (46,50) selection bias (53), and no sample size justification (47). Marques et al, 2008 was considered as high risk of bias because of selection bias, no sample size justification, inappropriate exposure measurements, and inadequate adjustment for confounders (52). Among the ten cross- sectional studies assessing direct fish consumption, three were rated as low risk (20,30,55) four as medium (37,31,44,49) and three as high risk of bias (28,29,51). The main reasons for medium risk of bias were no sample size justification (44), selection bias (31), and over-extrapolation of results (27,49). Studies were considered high risk of bias due to inappropriate exposure measurements (28,29,51) inadequate adjustment for confounders (28,29,51) and inadequate reporting on recruitment (28,29).

*Risk of Bias (RoB) in studies investigating indirect fish consumption*

The cross-sectional study design was employed in the remaining 12 studies. Four were appraised as low risk of bias (18,20,23,33) six as medium (19,21,24,32,36,45) and two as high risk of bias (40, 41). Studies were ranked as medium risk of bias because of inappropriate classification of exposure (32) and outcome measurements (19,32) inadequate adjustment for confounders (21,24) and selection bias (35,45). Both high risk of bias studies (40,41) had inadequate reporting on recruitment procedures; one study inadequately adjusted for confounders (41) and another over-extrapolated their results (40).

Of the 14 studies that measured indirect fish consumption, two were longitudinal (Table 4). One was considered as low risk of bias (48) while the other was considered as medium risk of bias (54) due to no sample size justification and inadequate adjustment for confounders.
